# Differential expression of olive flounder (*Paralichthys olivaceus*) transcriptome during viral hemorrhagic septicemia virus (VHSV) infection at warmer and colder temperature

**DOI:** 10.1016/j.dib.2018.06.085

**Published:** 2018-06-28

**Authors:** Ji-Min Jeong, Joseph Jeswin, Jin-Sol Bae, Chan-Il Park

**Affiliations:** Department of Marine Biology & Aquaculture, College of Marine Science, Gyeongsang National University, 455, Tongyeong 650-160, Republic of Korea

**Keywords:** *Paralichthys olivaceus*, Olive flounder, Transcriptome, RNA-Seq, Viral hemorrhagic septicemia virus (VHSV), Head kidney

## Abstract

The data presented here are related to the research article entitled “Temperature-dependent immune response of olive flounder (*Paralichthys olivaceus*) infected with viral hemorrhagic septicemia virus (VHSV)” [1]. In the cited article, we sequenced the whole transcriptome of the olive flounder using Illumina RNA-Seq. Differentially expressed genes (DEG) analysis of VHSV infected head kidney samples showed perturbations in gene expression. Herein we made a comparison of DEGs at early stage of VHSV infection of olive flounder (4 h post infection) in colder (13 °C) and warmer (20 °C) temperatures. The analysis of signaling pathways showed that several major immune pathways were altered. The gene ontology terms associated with the genes differentially expressed are also presented.

**Specifications Table**

**Table:** 

Subject area	Biology
More specific subject area	Transcriptomics
Type of data	Transcriptome sequences
How data was acquired	Illumina HiSeq. 2500
Data format	Raw data (FASTQ)
Experimental factors	Olive flounder were infected with VSHV at 13 and 20 °C. Samples of head kidney was collected at 4 h post infection
Experimental features	DEGs of olive flounder at 13 and 20 °C post VHSV infection
Sample source location	National Institute of Fisheries Science, Busan, South Korea
Data accessibility	Data is available in the article and at: https://www.ncbi.nlm.nih.gov//bioproject/PRJNA379500

**Value of the data**

● The information of unigenes expressed at colder and warmer temperature helps us to know how the response of host transcriptome varies with respect to surrounding environment.
● The comparison of modulated genes during VHSV infection at 13 and 20 °C helps for management measures in olive flounder aquaculture.
● The identification of affected signaling pathways in the head kidney of VHSV infected olive flounder sheds new light on the investigation of disease pathogenesis and for novel treatment targets.

## 1 Data

**Fig. 1:**
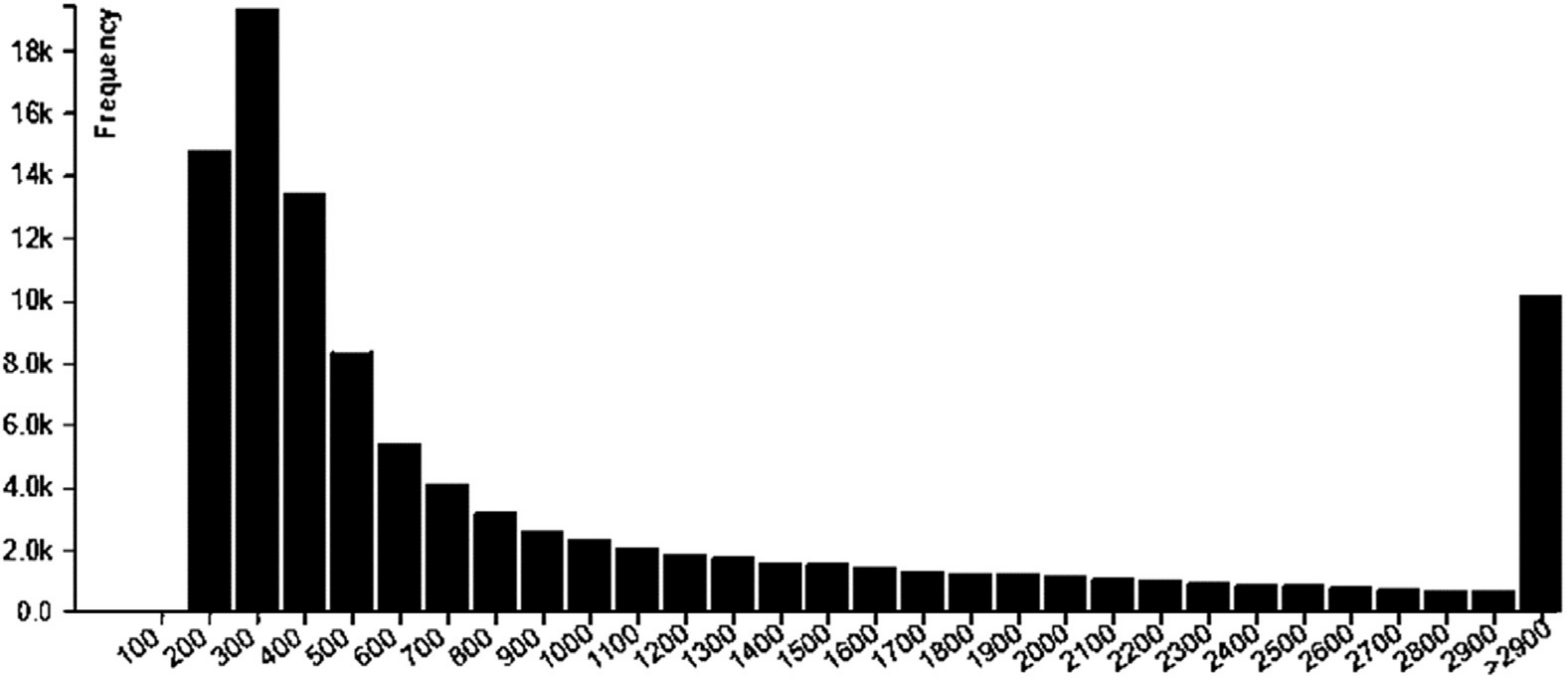
Analysis of sequencing reads assembly quality. Contig length distribution of Trinity assembly for olive flounder.

**Fig. 2:**
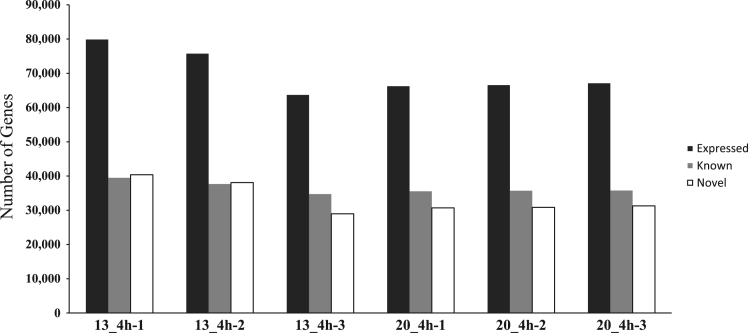
Unigenes expressed during VHSV infection in olive flounder at 13 °C and 20 °C. Three samples were screened at 4 hpi.

**Fig. 3:**
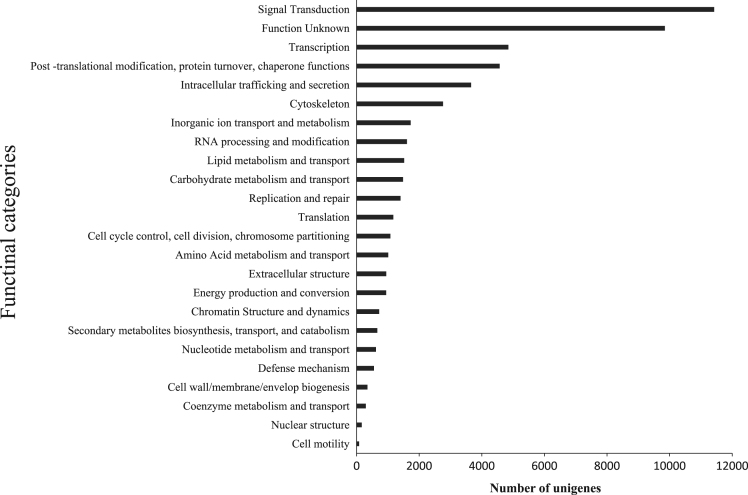
EggNOG classification assigned to annotated unigenes.

The data presented here are related to the research article entitled “*Temperature-dependent immune response of olive flounder (Paralichthys olivaceus) infected with viral hemorrhagic septicemia virus (VHSV)*” [1]. The olive flounder were challenged with VHSV at 13 °C and 20 °C, and DEG in the head kidney were analyzed. The whole transcriptome of olive flounder was sequenced using illumina RNA Seq. The quality of sequencing reads were assessed by contig length distribution of sequences and gene ontology functional analysis were conducted (Fig. 1, Supplementary table 1). Fig. 2 represents the number of unigenes which expressed during the infection period. All the unigenes were aligned to the eggNOG database and predicted the possible functions (Fig. 3). Table 1 describes the number of differentially expressed genes at 4 hours post infection of VHSV. The signaling pathways annotated in the transcriptome of olive flounder is shown in the Table 2 and Supplementary table. 2.

**Table 1:** Analysis of differentially expressed genes during VHSV infection in olive flounder at 13 °C and 20 °C. Here upregulation is Group 2 > Group 1 and downregulation is Group 2 < Group 1 (p-value < 0.05).

**Group 1**	**Group 2**	**Up regulated genes**	**Down regulated genes**
13 °C (4 h post infection)	20 °C (4 h post infection)	569	797

**Table 2:** Immune signalling pathways annotated in the olive flounder head kidney transcriptome.

**KEGG ID**	**KEGG Description**	**Number of Unigenes**
Ko04151	PI3K-Akt signaling pathway [PATH:ko04151]	296
Ko04010	MAPK signaling pathway [PATH:ko04010]	200
Ko04014	Ras signaling pathway [PATH:ko04014]	193
Ko04140	Autophagy - animal [PATH:ko04140]	175
Ko04024	cAMP signaling pathway [PATH:ko04024]	166
Ko04062	Chemokine signaling pathway [PATH:ko04062]	164
Ko04144	Endocytosis [PATH:ko04144]	160
Ko04152	AMPK signaling pathway [PATH:ko04152]	146
Ko04210	Apoptosis [PATH:ko04210]	125
Ko04668	TNF signaling pathway [PATH:ko04668]	120
Ko04150	mTOR signaling pathway [PATH:ko04150]	116
Ko04310	Wnt signaling pathway [PATH:ko04310]	107
Ko04625	C-type lectin receptor signaling pathway [PATH:ko04625]	105
Ko04620	Toll-like receptor signaling pathway [PATH:ko04620]	100
Ko04120	Ubiquitin mediated proteolysis [PATH:ko04120]	99
Ko04660	T cell receptor signaling pathway [PATH:ko04660]	99
Ko04064	NF-kappa B signaling pathway [PATH:ko04064]	77
Ko04060	Cytokine-cytokine receptor interaction [PATH:ko04060]	76
Ko04630	Jak-STAT signaling pathway [PATH:ko04630]	69
Ko04657	IL-17 signaling pathway [PATH:ko04657]	68
Ko04662	B cell receptor signaling pathway [PATH:ko04662]	67
Ko04624	Toll and Imd signaling pathway [PATH:ko04624]	40
Ko04610	Complement and coagulation cascades [PATH:ko04610]	21
Ko04217	TNF signaling pathway [PATH:ko04668]	13
Ko05220	Wnt signaling pathway [PATH:ko04310]	13
Ko04060	Chemokine signaling pathway [PATH:ko04062]	10
Ko04510	PI3K-Akt signaling pathway [PATH:ko04151]	9
Ko00970	Aminoacyl-tRNA biosynthesis [PATH:ko00970]	9
Ko05203	MAPK signaling pathway [PATH:ko04010]	8
Ko00051	AMPK signaling pathway [PATH:ko04152]	8
Ko04068	Chemokine signaling pathway [PATH:ko04062]	8
Ko04150	AMPK signaling pathway [PATH:ko04152]	8
Ko04270	MAPK signaling pathway [PATH:ko04010]	8
Ko04380	NF-kappa B signaling pathway [PATH:ko04064]	8
Ko04150	PI3K-Akt signaling pathway [PATH:ko04151]	8
Ko04150	MAPK signaling pathway [PATH:ko04010]	6
Ko04013	Toll and Imd signaling pathway [PATH:ko04624]	6
Ko04727	AMPK signaling pathway [PATH:ko04152]	6
Ko05152	C-type lectin receptor signaling pathway [PATH:ko04625]	5
Ko00563	NOD-like receptor signaling pathway [PATH:ko04621]	5
Ko05012	Ubiquitin mediated proteolysis [PATH:ko04120]	5
Ko04010	Ras signaling pathway [PATH:ko04014]	5
Ko04666	Endocytosis [PATH:ko04144]	5
Ko04611	Complement and coagulation cascades [PATH:ko04610]	5
Ko04010	PI3K-Akt signaling pathway [PATH:ko04151]	4
Ko04370	MAPK signaling pathway [PATH:ko04010]	4
Ko04510	Chemokine signaling pathway [PATH:ko04062]	4
Ko04144	Ubiquitin mediated proteolysis [PATH:ko04120]	4
Ko04510	MAPK signaling pathway [PATH:ko04010]	4
Ko05133	Complement and coagulation cascades [PATH:ko04610]	4

## 2 Experimental design, materials and methods

### 2.1 Experimental animals

Olive flounder of average weight 39.7 g were purchased from a commercial fish farm (Geoje Island) without any history of VHSV. Animals were maintained at 11–13 °C, and acclimated for one week.

### 2.2 Viral challenge, preparation of mRNA library and RNA seq

The fish of each groups were intraperitoneally injected (Isolate: FDC-VHS2014-5) with a VHSV dose of 1×10^4^ TCID_50_ per fish or control media in 0.1 ml and acclimatized at 13 and 20 °C, separately. Total RNA was isolated from the head kidney of three individual VHSV-infected olive flounder cultured on 13 and 20 °C. At 4 h post infection, three individuals were randomly collected from each group and head kidneys were excised for gene expression analysis. Kidney tissue samples were stored at −80 °C until RNA isolation. Total RNA was isolated using a standard Trizol extraction protocol (Invitrogen, Germany) according to the manufacturer׳s instructions. The concentration and integrity of the RNA were assessed with a Thermo Scientific NanoDrop 8000 Spectrophotometer and Agilent 2100 Bioanalyzer, respectively. RNA with an OD_260/280_≥1.8 and an RNA integrity number ≥7.0 was used in subsequent experiments. Equal amounts of high quality RNA from each sample were then used separately for cDNA synthesis and sequencing. The cDNA library was prepared with ~1.0 μg of total RNA following manufacturer׳s recommendations of TrueSeq RNA library Preparation Kit (Illumina, USA). The library was then amplified, and the final library yielded ~ 500 ng of cDNA with an average fragment size of ~ 350 bp. The resulting cDNA libraries were then paired-end sequenced (2 × 100 bp) with HiSeq. 2500 platform (Illumina, USA). All sequencing reads were deposited in the NCBI Sequence Read Archive (SRA) under the accession number SRP102673.

### 2.3 Transcriptome de novo assembly, annotation and differential expression

The raw reads of fastq format were undergone pre-processing and high quality sequences were subject to de novo assembly using Trinity software [2]. The assembled unigenes were BLASTX mapped against NCBI non redundant protein and swiss-prot databases. Gene ontology (GO) terms were assigned to each unigene based on the GO terms annotated to its corresponding homologs. The differential expression of unigenes were analyzed by aligning individual sample reads with reference transcriptome using Bowtie2 [3]. Moreover, unigenes were assigned to biochemical pathways according to the Kyoto Encyclopedia of Genes and Genomes (KEGG) database using BLASTX, followed by retrieving KEGG Orthology (KO) information. Additionally, the Clusters of Orthologous Groups (COG) screening was performed using the eggNOG database [4].
